# Crystal structure of ethyl 5-(3-fluoro­phen­yl)-2-[(4-fluoro­phen­yl)methyl­idene]-7-methyl-3-oxo-2*H*,3*H*,5*H*-[1,3]thia­zolo[3,2-*a*]pyrimidine-6-carboxyl­ate

**DOI:** 10.1107/S1600536814023010

**Published:** 2014-10-24

**Authors:** M. S. Krishnamurthy, H. Nagarajaiah, Noor Shahina Begum

**Affiliations:** a Department of Studies in Chemistry, Bangalore University, Bangalore 560 001, Karnataka, India

**Keywords:** crystal structure, pyrimidine, thia­zole, hydrogen bonds, π–π stacking inter­actions

## Abstract

In the title mol­ecule, C_23_H_18_F_2_N_2_O_3_S, the pyrimidine ring is in a half-chair conformation and the 3-fluoro­phenyl group is in the axial position. The thia­zole ring (r.m.s. deviation = 0.0252 Å) forms dihedral angles of 84.8 (7) and 9.6 (7)° with the 3-fluoro-substituted and 4-fluoro-substituted benzene rings, respectively. In the crystal, weak C—H⋯F and C—H⋯O hydrogen bonds connect mol­ecules, forming zigzag chains along the *b* axis. In addition π–π stacking inter­actions with a centroid–centroid distance of 3.7633 (9) Å connect these chains into ladders *via* inversion-related 4-fluoro­phenyl groups.

## Related literature   

For the pharmacological properties of pyrimidine derivatives, see: Alam *et al.* (2010 ▶); Kulakov *et al.* (2009 ▶); Ashok *et al.* (2007 ▶). For examples of compounds with weak inter­molecular inter­actions, see: Prasanna & Guru Row (2001 ▶); Yamazaki *et al.* (2009 ▶). For related structures, see: Fischer *et al.* (2007 ▶); Zhao *et al.* (2011 ▶); Nagarajaiah & Begum (2011 ▶).
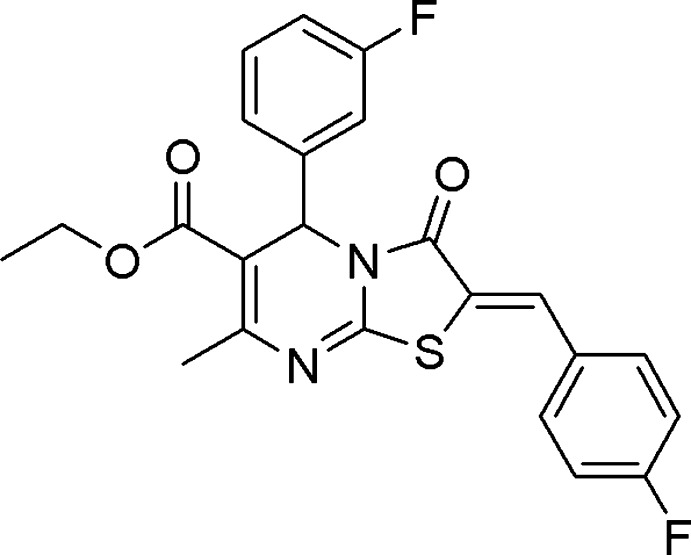


## Experimental   

### Crystal data  

C_23_H_18_F_2_N_2_O_3_S
*M* *_r_* = 440.45
Monoclinic, 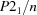

*a* = 9.4358 (5) Å
*b* = 10.7862 (6) Å
*c* = 20.2246 (11) Å
β = 92.159 (1)°
*V* = 2056.93 (19) Å^3^
*Z* = 4
Mo *K*α radiation
μ = 0.20 mm^−1^
*T* = 100 K
0.18 × 0.16 × 0.16 mm

### Data collection  

Bruker SMART APEX diffractometer
Absorption correction: multi-scan (*SADABS*; Bruker, 1998 ▶) *T*_min_ = 0.964, *T*_max_ = 0.968
15631 measured reflections
4480 independent reflections
3890 reflections with *I* > 2σ(*I*)
*R*_int_ = 0.026

### Refinement  

*R*[*F* ^2^ > 2σ(*F* ^2^)] = 0.040
*wR*(*F* ^2^) = 0.104
*S* = 1.06
4480 reflections
282 parameters
H-atom parameters constrained
Δρ_max_ = 0.44 e Å^−3^
Δρ_min_ = −0.23 e Å^−3^

### 

Data collection: *SMART* (Bruker, 1998 ▶); cell refinement: *SAINT-Plus* (Bruker, 1998 ▶); data reduction: *SAINT-Plus*; program(s) used to solve structure: *SHELXS97* (Sheldrick, 2008 ▶); program(s) used to refine structure: *SHELXL97* (Sheldrick, 2008 ▶); molecular graphics: *ORTEP-3 for Windows* (Farrugia, 2012 ▶) and *CAMERON* (Watkin *et al.*, 1996 ▶); software used to prepare material for publication: *WinGX* (Farrugia, 2012 ▶).

## Supplementary Material

Crystal structure: contains datablock(s) global, I. DOI: 10.1107/S1600536814023010/lh5711sup1.cif

Structure factors: contains datablock(s) I. DOI: 10.1107/S1600536814023010/lh5711Isup2.hkl

Supporting information file. DOI: 10.1107/S1600536814023010/lh5711Isup3.cml

**Fig. 1.**
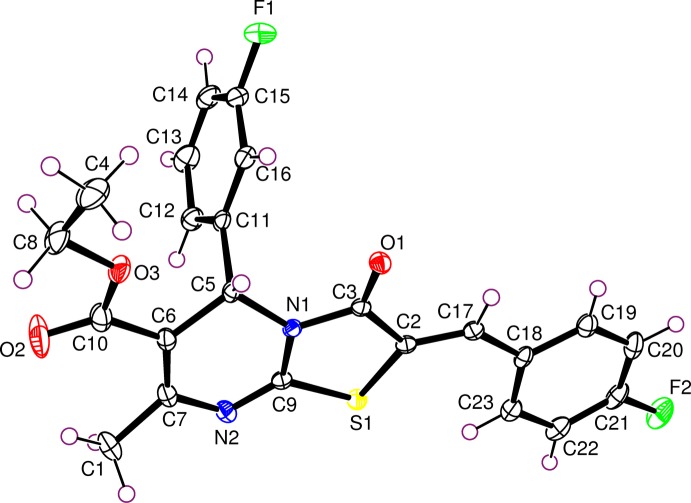
The molecular structure of the title compound with displacement ellipsoids drawn at the 50% probability level. H atoms are presented as small spheres of arbitrary radius.

. DOI: 10.1107/S1600536814023010/lh5711fig1.tif
The mol­ecular structure of the title compound with displacement ellipsoids drawn at the 50% probability level. H atoms are presented as small spheres of arbitrary radius.

**Fig. 2.**
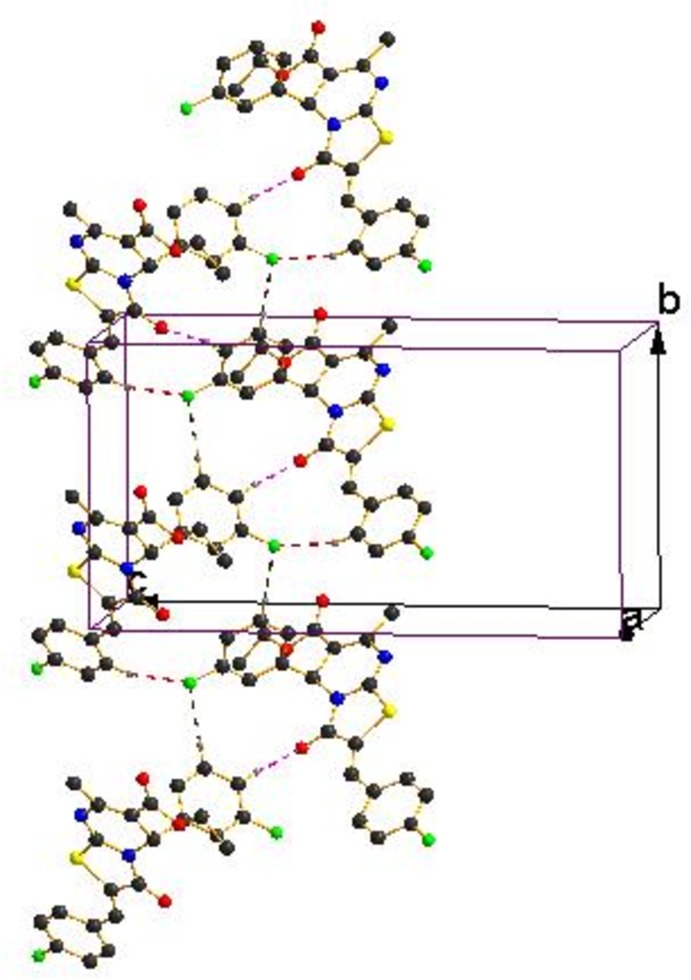
Part of the crystal structure showing intermolecular interactions with dotted lines. H-atoms not involved in hydrogen bonds have been excluded.

. DOI: 10.1107/S1600536814023010/lh5711fig2.tif
Part of the crystal structure showing inter­molecular inter­actions with dotted lines. H-atoms not involved in hydrogen bonds have been excluded.

CCDC reference: 1029845

Additional supporting information:  crystallographic information; 3D view; checkCIF report

## Figures and Tables

**Table 1:** Hydrogen-bond geometry (, )

*D*H*A*	*D*H	H*A*	*D* *A*	*D*H*A*
C19H19F1^i^	0.95	2.53	3.437(2)	159
C13H13F1^ii^	0.95	2.56	3.513(2)	178
C14H14O1^ii^	0.95	2.36	3.303(2)	172

Symmetry codes: (i) 
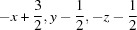
; (ii) 
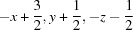
.

## supplementary crystallographic information

### S1. Comment

Pyrimidine derivatives are of interest because of their useful biological and therapeutic activities (Ashok *et al.*, 2007). The presence of both pyrimidine and thiazole rings results in enhanced activity (Alam *et al.*, 2010; Kulakov *et al.*, 2009). Intermolecular interactions of the type C—H···F and C—F···π can supply both directional and actively favorable pathways to hold the molecules together in the crystalline lattice that offer additional stability (Prasanna & Guru Row, 2001; Yamazaki *et al.*, 2009). Herein, we report the crystal structure of the title compound.

The molecular structure of the title compound is shown in Fig. 1. The 3-fluoro phenyl ring adopts a *syn* periplanar conformation with respect to C5—H5 bond of the pyrimidine ring. The pyrimidine ring is in a half-chair conformation with atoms N1 and C5 displaced by -0.110 (1) and 0.168 (1) Å from the mean plane of the other four atoms (N2/C6/C7/C9 with a maximun deviation of 0.042 (1)Å for both atoms N2 and C7). The 3-fluorophenyl group is in the axial position. The thiazole ring (r.m.s. deviation = 0.0252 Å) forms dihedral angles of 84.8 (7) and 9.6 (7)° with the 3-fluoro-substituted and 4-fluoro-substituted benzene rings respectively. The exocyclic ester group at C6 adopts *cis* orientation with respect to C6═C7 double bond. The bond and angles in the title compound agree with the corresponding bond distances and angles reported in related compounds (Fischer *et al.*, 2007; Zhao *et al.*, 2011; Nagarajaiah & Begum, 2011). In the crystal, weak C—H···F and C—H···O hydrogen bonds connect molecules forming zigzag chains along the *b* axis (Fig. 2). In addition π–π stacking interactions with a centroid–centroid distance of 3.7633 (9)Å connect these chains into ladders via inversion-related 4-fluorophenyl groups.

### S2. Experimental

A mixture of 4-(3-fluoro-phenyl)-6-methyl-2-thioxo-1,2,3,4-tetrahydro- pyrimidine-5-carboxylic acid ethyl ester (10 mmol), chloroacetic acid (10 mmol), 4-fluorobenzaldehyde (10 mmol) and sodium acetate (1.5 g) was taken in a mixture of glacial acetic acid and acetic anhydride (25 ml; 1:1) and refluxed for 8–10 h until the TLC assay indicated that the reaction was complete. The reaction mixture was concentrated and the solid thus obtained was filtered and recrystallized from ethyl acetate to get the title compound (76% yield, mp 460 K). The compound was recrystallized by slow evaporation from dimethylformamide (DMF) solvent, yielding pale yellow single crystals suitable for X-ray diffraction studies.

### S3. Refinement

The H atoms were placed at calculated positions in the riding model approximation with C—H = 0.95–1.00 Å with *U*_iso_(H) = 1.5*U*_eq_(C) for methyl H atoms and *U*_iso_(H) = 1.2*U*_eq_(C) for other hydrogen atoms.

### Crystal data

**Table:**

C_23_H_18_F_2_N_2_O_3_S	*F*(000) = 912
*M**_r_* = 440.45	*D*_x_ = 1.422 Mg m^−^^3^
Monoclinic, *P*2_1_/*n*	Mo *K*α radiation, λ = 0.71073 Å
Hall symbol: -P 2yn	Cell parameters from 4480 reflections
*a* = 9.4358 (5) Å	θ = 2.1–27.0°
*b* = 10.7862 (6) Å	µ = 0.20 mm^−^^1^
*c* = 20.2246 (11) Å	*T* = 100 K
β = 92.159 (1)°	Block, yellow
*V* = 2056.93 (19) Å^3^	0.18 × 0.16 × 0.16 mm
*Z* = 4	

### Data collection

**Table:**

Bruker SMART APEX diffractometer	4480 independent reflections
Radiation source: fine-focus sealed tube	3890 reflections with *I* > 2σ(*I*)
Graphite monochromator	*R*_int_ = 0.026
ω scans	θ_max_ = 27.0°, θ_min_ = 2.1°
Absorption correction: multi-scan (*SADABS*; Bruker, 1998)	*h* = −11→12
*T*_min_ = 0.964, *T*_max_ = 0.968	*k* = −13→11
15631 measured reflections	*l* = −25→25

### Refinement

**Table:**

Refinement on *F*^2^	Primary atom site location: structure-invariant direct methods
Least-squares matrix: full	Secondary atom site location: difference Fourier map
*R*[*F*^2^ > 2σ(*F*^2^)] = 0.040	Hydrogen site location: inferred from neighbouring sites
*wR*(*F*^2^) = 0.104	H-atom parameters constrained
*S* = 1.06	*w* = 1/[σ^2^(*F*_o_^2^) + (0.0546*P*)^2^ + 0.9996*P*] where *P* = (*F*_o_^2^ + 2*F*_c_^2^)/3
4480 reflections	(Δ/σ)_max_ = 0.001
282 parameters	Δρ_max_ = 0.44 e Å^−^^3^
0 restraints	Δρ_min_ = −0.23 e Å^−^^3^

### Special details

**Table:**

Geometry. All s.u.'s (except the s.u. in the dihedral angle between two l.s. planes) are estimated using the full covariance matrix. The cell s.u.'s are taken into account individually in the estimation of s.u.'s in distances, angles and torsion angles; correlations between s.u.'s in cell parameters are only used when they are defined by crystal symmetry. An approximate (isotropic) treatment of cell s.u.'s is used for estimating s.u.'s involving l.s. planes.
Refinement. Refinement of *F*^2^ against ALL reflections. The weighted *R*-factor *wR* and goodness of fit *S* are based on *F*^2^, conventional *R*-factors *R* are based on *F*, with *F* set to zero for negative *F*^2^. The threshold expression of *F*^2^ > 2σ(*F*^2^) is used only for calculating *R*-factors(gt) *etc*. and is not relevant to the choice of reflections for refinement. *R*-factors based on *F*^2^ are statistically about twice as large as those based on *F*, and *R*- factors based on ALL data will be even larger.

### Fractional atomic coordinates and isotropic or equivalent isotropic displacement parameters (Å^2^)

**Table:**

	*x*	*y*	*z*	*U*_iso_*/*U*_eq_	
S1	0.88687 (4)	0.19204 (3)	0.034764 (17)	0.01589 (11)	
F2	0.38224 (11)	−0.20262 (10)	0.14390 (5)	0.0344 (3)	
N1	1.02387 (12)	0.21900 (11)	−0.07291 (6)	0.0130 (2)	
O1	0.95802 (11)	0.05123 (9)	−0.13566 (5)	0.0168 (2)	
O3	1.32012 (11)	0.35754 (11)	−0.18945 (5)	0.0234 (3)	
N2	1.08494 (13)	0.36112 (12)	0.01251 (6)	0.0178 (3)	
F1	0.83639 (12)	0.28603 (11)	−0.33642 (5)	0.0364 (3)	
C6	1.20222 (15)	0.37625 (13)	−0.09169 (7)	0.0168 (3)	
C2	0.86191 (15)	0.08022 (13)	−0.02757 (7)	0.0136 (3)	
C17	0.77697 (14)	−0.01947 (13)	−0.02952 (7)	0.0145 (3)	
H17	0.7813	−0.0677	−0.0687	0.017*	
C9	1.01253 (15)	0.27081 (13)	−0.01116 (7)	0.0138 (3)	
C11	0.99691 (15)	0.34259 (14)	−0.17420 (7)	0.0160 (3)	
C5	1.10183 (15)	0.28309 (13)	−0.12475 (7)	0.0144 (3)	
H5	1.1592	0.2205	−0.1485	0.017*	
C18	0.67908 (15)	−0.06573 (13)	0.01880 (7)	0.0157 (3)	
C21	0.48097 (17)	−0.15811 (16)	0.10318 (8)	0.0240 (3)	
C7	1.19145 (15)	0.40940 (13)	−0.02781 (8)	0.0175 (3)	
C16	0.96568 (17)	0.28569 (15)	−0.23478 (8)	0.0202 (3)	
H16	1.0110	0.2107	−0.2466	0.024*	
C3	0.94988 (14)	0.11093 (13)	−0.08497 (7)	0.0135 (3)	
C20	0.48715 (17)	−0.20784 (15)	0.04067 (9)	0.0236 (3)	
H20	0.4247	−0.2726	0.0267	0.028*	
C19	0.58648 (16)	−0.16121 (14)	−0.00123 (8)	0.0195 (3)	
H19	0.5921	−0.1946	−0.0445	0.023*	
C23	0.67034 (16)	−0.01971 (15)	0.08349 (8)	0.0202 (3)	
H23	0.7331	0.0442	0.0983	0.024*	
C15	0.86709 (17)	0.34154 (16)	−0.27702 (7)	0.0243 (4)	
C10	1.31041 (16)	0.42857 (15)	−0.13541 (8)	0.0235 (3)	
O2	1.37971 (16)	0.52124 (13)	−0.12605 (8)	0.0448 (4)	
C13	0.83007 (17)	0.50531 (16)	−0.20222 (9)	0.0266 (4)	
H13	0.7836	0.5800	−0.1908	0.032*	
C12	0.92960 (16)	0.45299 (15)	−0.15833 (8)	0.0206 (3)	
H12	0.9519	0.4926	−0.1173	0.025*	
C1	1.28965 (17)	0.49369 (16)	0.01043 (9)	0.0253 (4)	
H1A	1.3665	0.5199	−0.0177	0.038*	
H1B	1.3296	0.4498	0.0493	0.038*	
H1C	1.2373	0.5667	0.0248	0.038*	
C22	0.57151 (17)	−0.06610 (15)	0.12598 (8)	0.0235 (3)	
H22	0.5663	−0.0352	0.1698	0.028*	
C14	0.79808 (17)	0.44933 (16)	−0.26257 (8)	0.0265 (4)	
H14	0.7303	0.4846	−0.2930	0.032*	
C8	1.41942 (18)	0.39730 (18)	−0.23844 (9)	0.0309 (4)	
H8A	1.5130	0.4160	−0.2170	0.037*	
H8B	1.3840	0.4728	−0.2615	0.037*	
C4	1.4318 (2)	0.29285 (19)	−0.28635 (9)	0.0358 (4)	
H4A	1.4729	0.2205	−0.2635	0.054*	
H4B	1.4931	0.3178	−0.3221	0.054*	
H4C	1.3375	0.2717	−0.3050	0.054*	

### Atomic displacement parameters (Å^2^)

**Table:**

	*U*^11^	*U*^22^	*U*^33^	*U*^12^	*U*^13^	*U*^23^
S1	0.01805 (19)	0.01604 (19)	0.01374 (18)	−0.00094 (13)	0.00276 (13)	−0.00157 (13)
F2	0.0247 (5)	0.0389 (6)	0.0406 (6)	−0.0027 (4)	0.0143 (4)	0.0164 (5)
N1	0.0131 (6)	0.0139 (6)	0.0121 (5)	−0.0001 (4)	0.0003 (4)	0.0014 (4)
O1	0.0185 (5)	0.0175 (5)	0.0147 (5)	−0.0020 (4)	0.0024 (4)	−0.0026 (4)
O3	0.0197 (6)	0.0269 (6)	0.0238 (6)	−0.0057 (5)	0.0057 (4)	0.0064 (5)
N2	0.0167 (6)	0.0160 (6)	0.0206 (6)	−0.0004 (5)	−0.0012 (5)	−0.0029 (5)
F1	0.0456 (7)	0.0419 (6)	0.0206 (5)	−0.0067 (5)	−0.0127 (5)	0.0051 (4)
C6	0.0127 (7)	0.0136 (7)	0.0238 (7)	−0.0002 (5)	−0.0023 (6)	0.0026 (6)
C2	0.0131 (6)	0.0147 (7)	0.0131 (6)	0.0026 (5)	−0.0002 (5)	0.0006 (5)
C17	0.0130 (7)	0.0154 (7)	0.0152 (7)	0.0028 (5)	0.0000 (5)	0.0014 (5)
C9	0.0133 (6)	0.0139 (6)	0.0143 (6)	0.0030 (5)	−0.0002 (5)	0.0009 (5)
C11	0.0126 (7)	0.0176 (7)	0.0178 (7)	−0.0033 (5)	0.0008 (5)	0.0060 (6)
C5	0.0120 (6)	0.0147 (7)	0.0167 (7)	−0.0012 (5)	0.0022 (5)	0.0025 (5)
C18	0.0132 (7)	0.0133 (7)	0.0207 (7)	0.0033 (5)	0.0014 (5)	0.0053 (6)
C21	0.0165 (7)	0.0255 (8)	0.0306 (8)	0.0042 (6)	0.0085 (6)	0.0141 (7)
C7	0.0125 (7)	0.0124 (7)	0.0273 (8)	0.0010 (5)	−0.0025 (6)	−0.0005 (6)
C16	0.0215 (8)	0.0192 (7)	0.0198 (7)	−0.0027 (6)	0.0003 (6)	0.0050 (6)
C3	0.0116 (6)	0.0144 (7)	0.0142 (6)	0.0012 (5)	−0.0017 (5)	0.0020 (5)
C20	0.0157 (7)	0.0179 (8)	0.0373 (9)	−0.0015 (6)	0.0006 (6)	0.0084 (7)
C19	0.0164 (7)	0.0169 (7)	0.0252 (8)	0.0022 (6)	0.0001 (6)	0.0041 (6)
C23	0.0191 (7)	0.0193 (7)	0.0222 (7)	0.0007 (6)	0.0021 (6)	0.0044 (6)
C15	0.0247 (8)	0.0309 (9)	0.0169 (7)	−0.0095 (7)	−0.0038 (6)	0.0071 (7)
C10	0.0166 (7)	0.0215 (8)	0.0323 (9)	−0.0025 (6)	0.0007 (6)	0.0042 (7)
O2	0.0439 (8)	0.0382 (8)	0.0535 (9)	−0.0277 (7)	0.0159 (7)	−0.0068 (7)
C13	0.0195 (8)	0.0275 (9)	0.0329 (9)	0.0055 (6)	0.0019 (7)	0.0090 (7)
C12	0.0186 (7)	0.0208 (8)	0.0226 (7)	0.0010 (6)	0.0010 (6)	0.0039 (6)
C1	0.0181 (8)	0.0214 (8)	0.0361 (9)	−0.0035 (6)	−0.0021 (7)	−0.0083 (7)
C22	0.0237 (8)	0.0254 (8)	0.0217 (8)	0.0044 (6)	0.0059 (6)	0.0072 (6)
C14	0.0171 (7)	0.0336 (9)	0.0285 (8)	−0.0019 (6)	−0.0037 (6)	0.0160 (7)
C8	0.0229 (8)	0.0393 (10)	0.0311 (9)	−0.0082 (7)	0.0091 (7)	0.0114 (8)
C4	0.0371 (10)	0.0491 (12)	0.0219 (8)	−0.0069 (9)	0.0088 (7)	0.0084 (8)

### Geometric parameters (Å, º)

**Table:**

S1—C9	1.7531 (14)	C21—C22	1.378 (2)
S1—C2	1.7542 (14)	C7—C1	1.493 (2)
F2—C21	1.3544 (17)	C16—C15	1.378 (2)
N1—C3	1.3758 (18)	C16—H16	0.9500
N1—C9	1.3761 (18)	C20—C19	1.382 (2)
N1—C5	1.4753 (17)	C20—H20	0.9500
O1—C3	1.2154 (17)	C19—H19	0.9500
O3—C10	1.341 (2)	C23—C22	1.385 (2)
O3—C8	1.4538 (18)	C23—H23	0.9500
N2—C9	1.2728 (19)	C15—C14	1.370 (3)
N2—C7	1.4171 (19)	C10—O2	1.206 (2)
F1—C15	1.3636 (19)	C13—C14	1.385 (2)
C6—C7	1.348 (2)	C13—C12	1.388 (2)
C6—C10	1.487 (2)	C13—H13	0.9500
C6—C5	1.519 (2)	C12—H12	0.9500
C2—C17	1.341 (2)	C1—H1A	0.9800
C2—C3	1.4897 (18)	C1—H1B	0.9800
C17—C18	1.4578 (19)	C1—H1C	0.9800
C17—H17	0.9500	C22—H22	0.9500
C11—C16	1.392 (2)	C14—H14	0.9500
C11—C12	1.393 (2)	C8—C4	1.493 (3)
C11—C5	1.5222 (19)	C8—H8A	0.9900
C5—H5	1.0000	C8—H8B	0.9900
C18—C19	1.401 (2)	C4—H4A	0.9800
C18—C23	1.405 (2)	C4—H4B	0.9800
C21—C20	1.376 (2)	C4—H4C	0.9800
			
C9—S1—C2	91.59 (7)	C19—C20—H20	120.8
C3—N1—C9	116.66 (12)	C20—C19—C18	121.37 (15)
C3—N1—C5	122.30 (11)	C20—C19—H19	119.3
C9—N1—C5	120.84 (12)	C18—C19—H19	119.3
C10—O3—C8	116.91 (13)	C22—C23—C18	120.98 (15)
C9—N2—C7	116.55 (12)	C22—C23—H23	119.5
C7—C6—C10	123.20 (14)	C18—C23—H23	119.5
C7—C6—C5	121.96 (13)	F1—C15—C14	118.29 (15)
C10—C6—C5	114.84 (13)	F1—C15—C16	118.06 (16)
C17—C2—C3	120.36 (13)	C14—C15—C16	123.65 (15)
C17—C2—S1	129.53 (11)	O2—C10—O3	123.27 (15)
C3—C2—S1	110.09 (10)	O2—C10—C6	127.00 (16)
C2—C17—C18	130.38 (13)	O3—C10—C6	109.72 (13)
C2—C17—H17	114.8	C14—C13—C12	120.42 (16)
C18—C17—H17	114.8	C14—C13—H13	119.8
N2—C9—N1	126.38 (13)	C12—C13—H13	119.8
N2—C9—S1	122.40 (11)	C13—C12—C11	120.25 (15)
N1—C9—S1	111.18 (10)	C13—C12—H12	119.9
C16—C11—C12	119.77 (14)	C11—C12—H12	119.9
C16—C11—C5	120.22 (13)	C7—C1—H1A	109.5
C12—C11—C5	120.00 (13)	C7—C1—H1B	109.5
N1—C5—C6	108.45 (11)	H1A—C1—H1B	109.5
N1—C5—C11	109.58 (11)	C7—C1—H1C	109.5
C6—C5—C11	112.94 (12)	H1A—C1—H1C	109.5
N1—C5—H5	108.6	H1B—C1—H1C	109.5
C6—C5—H5	108.6	C21—C22—C23	118.42 (15)
C11—C5—H5	108.6	C21—C22—H22	120.8
C19—C18—C23	118.12 (13)	C23—C22—H22	120.8
C19—C18—C17	117.51 (13)	C15—C14—C13	117.95 (15)
C23—C18—C17	124.36 (14)	C15—C14—H14	121.0
F2—C21—C20	118.31 (15)	C13—C14—H14	121.0
F2—C21—C22	118.93 (15)	O3—C8—C4	106.72 (14)
C20—C21—C22	122.76 (14)	O3—C8—H8A	110.4
C6—C7—N2	122.33 (13)	C4—C8—H8A	110.4
C6—C7—C1	126.18 (14)	O3—C8—H8B	110.4
N2—C7—C1	111.43 (13)	C4—C8—H8B	110.4
C15—C16—C11	117.95 (15)	H8A—C8—H8B	108.6
C15—C16—H16	121.0	C8—C4—H4A	109.5
C11—C16—H16	121.0	C8—C4—H4B	109.5
O1—C3—N1	123.41 (12)	H4A—C4—H4B	109.5
O1—C3—C2	126.46 (13)	C8—C4—H4C	109.5
N1—C3—C2	110.13 (12)	H4A—C4—H4C	109.5
C21—C20—C19	118.32 (15)	H4B—C4—H4C	109.5
C21—C20—H20	120.8		
			
C9—S1—C2—C17	179.32 (14)	C9—N1—C3—O1	−174.93 (13)
C9—S1—C2—C3	−2.35 (10)	C5—N1—C3—O1	10.2 (2)
C3—C2—C17—C18	−177.86 (13)	C9—N1—C3—C2	4.85 (17)
S1—C2—C17—C18	0.3 (2)	C5—N1—C3—C2	−169.99 (12)
C7—N2—C9—N1	−2.8 (2)	C17—C2—C3—O1	−2.5 (2)
C7—N2—C9—S1	174.64 (10)	S1—C2—C3—O1	178.97 (12)
C3—N1—C9—N2	170.94 (14)	C17—C2—C3—N1	177.70 (13)
C5—N1—C9—N2	−14.1 (2)	S1—C2—C3—N1	−0.80 (14)
C3—N1—C9—S1	−6.71 (15)	F2—C21—C20—C19	179.07 (13)
C5—N1—C9—S1	168.21 (10)	C22—C21—C20—C19	−1.5 (2)
C2—S1—C9—N2	−172.76 (13)	C21—C20—C19—C18	0.0 (2)
C2—S1—C9—N1	5.00 (11)	C23—C18—C19—C20	1.2 (2)
C3—N1—C5—C6	−164.71 (12)	C17—C18—C19—C20	−177.90 (14)
C9—N1—C5—C6	20.67 (17)	C19—C18—C23—C22	−1.0 (2)
C3—N1—C5—C11	71.60 (16)	C17—C18—C23—C22	178.08 (14)
C9—N1—C5—C11	−103.03 (14)	C11—C16—C15—F1	179.95 (13)
C7—C6—C5—N1	−13.70 (19)	C11—C16—C15—C14	−0.2 (2)
C10—C6—C5—N1	167.22 (12)	C8—O3—C10—O2	−0.4 (2)
C7—C6—C5—C11	107.95 (16)	C8—O3—C10—C6	178.65 (13)
C10—C6—C5—C11	−71.13 (16)	C7—C6—C10—O2	−16.7 (3)
C16—C11—C5—N1	−101.02 (15)	C5—C6—C10—O2	162.33 (17)
C12—C11—C5—N1	77.65 (16)	C7—C6—C10—O3	164.23 (14)
C16—C11—C5—C6	137.96 (14)	C5—C6—C10—O3	−16.70 (18)
C12—C11—C5—C6	−43.37 (17)	C14—C13—C12—C11	−0.9 (2)
C2—C17—C18—C19	169.68 (15)	C16—C11—C12—C13	1.0 (2)
C2—C17—C18—C23	−9.4 (2)	C5—C11—C12—C13	−177.68 (14)
C10—C6—C7—N2	178.12 (13)	F2—C21—C22—C23	−178.84 (14)
C5—C6—C7—N2	−0.9 (2)	C20—C21—C22—C23	1.7 (2)
C10—C6—C7—C1	−5.0 (2)	C18—C23—C22—C21	−0.4 (2)
C5—C6—C7—C1	175.96 (14)	F1—C15—C14—C13	−179.84 (14)
C9—N2—C7—C6	10.3 (2)	C16—C15—C14—C13	0.4 (2)
C9—N2—C7—C1	−166.99 (13)	C12—C13—C14—C15	0.2 (2)
C12—C11—C16—C15	−0.4 (2)	C10—O3—C8—C4	169.32 (15)
C5—C11—C16—C15	178.24 (13)		

### Hydrogen-bond geometry (Å, º)

**Table:**

*D*—H···*A*	*D*—H	H···*A*	*D*···*A*	*D*—H···*A*
C19—H19···F1^i^	0.95	2.53	3.437 (2)	159
C13—H13···F1^ii^	0.95	2.56	3.513 (2)	178
C14—H14···O1^ii^	0.95	2.36	3.303 (2)	172

Symmetry codes: (i) −*x*+3/2, *y*−1/2, −*z*−1/2; (ii) −*x*+3/2, *y*+1/2, −*z*−1/2.
