# Supplementary figures and images for: Crystal structure of ethyl 5-(3-fluoro­phen­yl)-2-[(4-fluoro­phen­yl)methyl­idene]-7-methyl-3-oxo-2H,3H,5H-[1,3]thia­zolo[3,2-a]pyrimidine-6-carboxyl­ate
Source: Acta Crystallogr Sect E Struct Rep Online. 2014 Oct 24;70(Pt 11):o1187–8. doi: 10.1107/S1600536814023010 (PMC4257247; doi:10.1107/S1600536814023010)

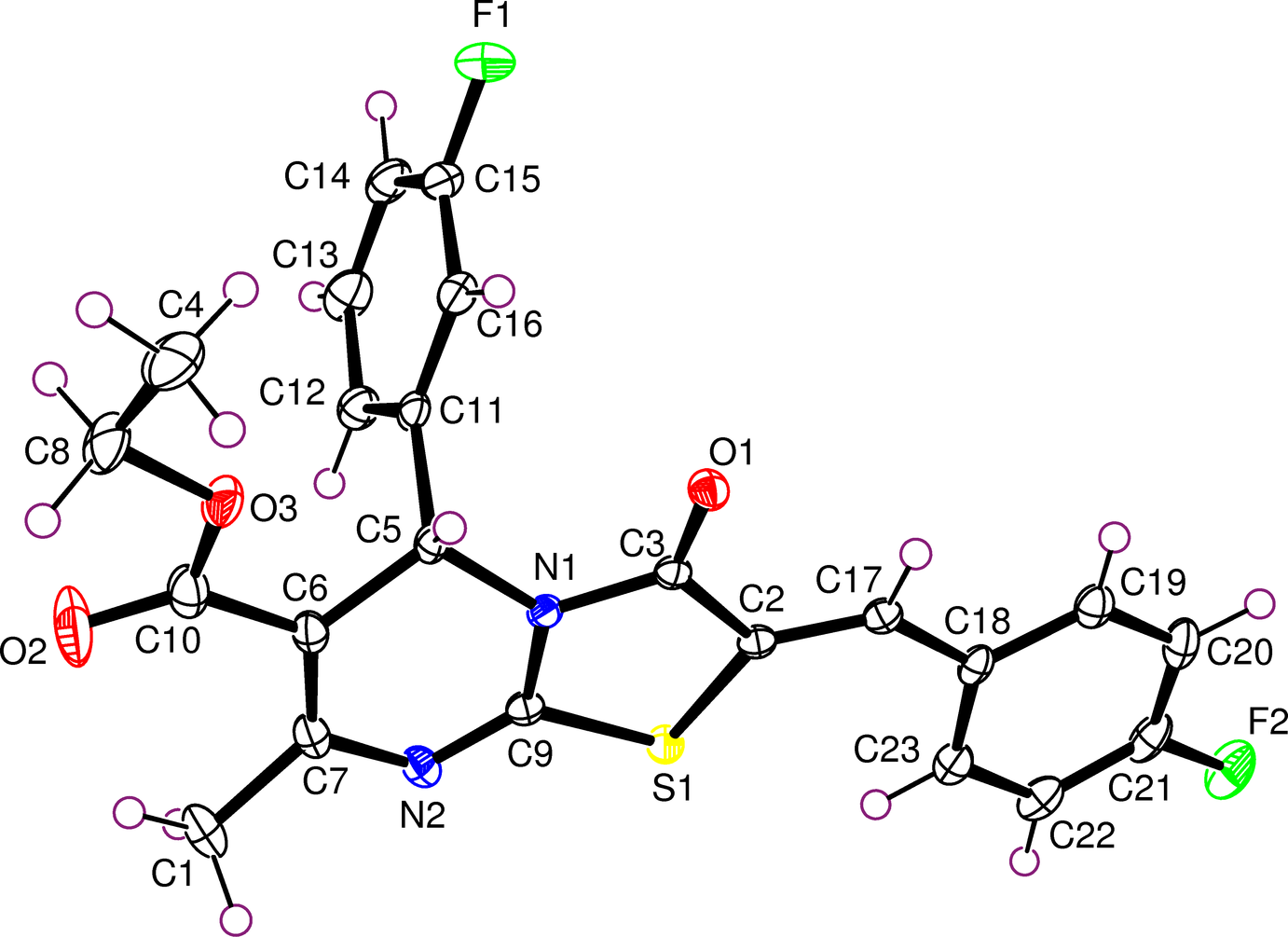

Supplement: Supplementary file 4 [file e-70-o1187-fig1.tif]

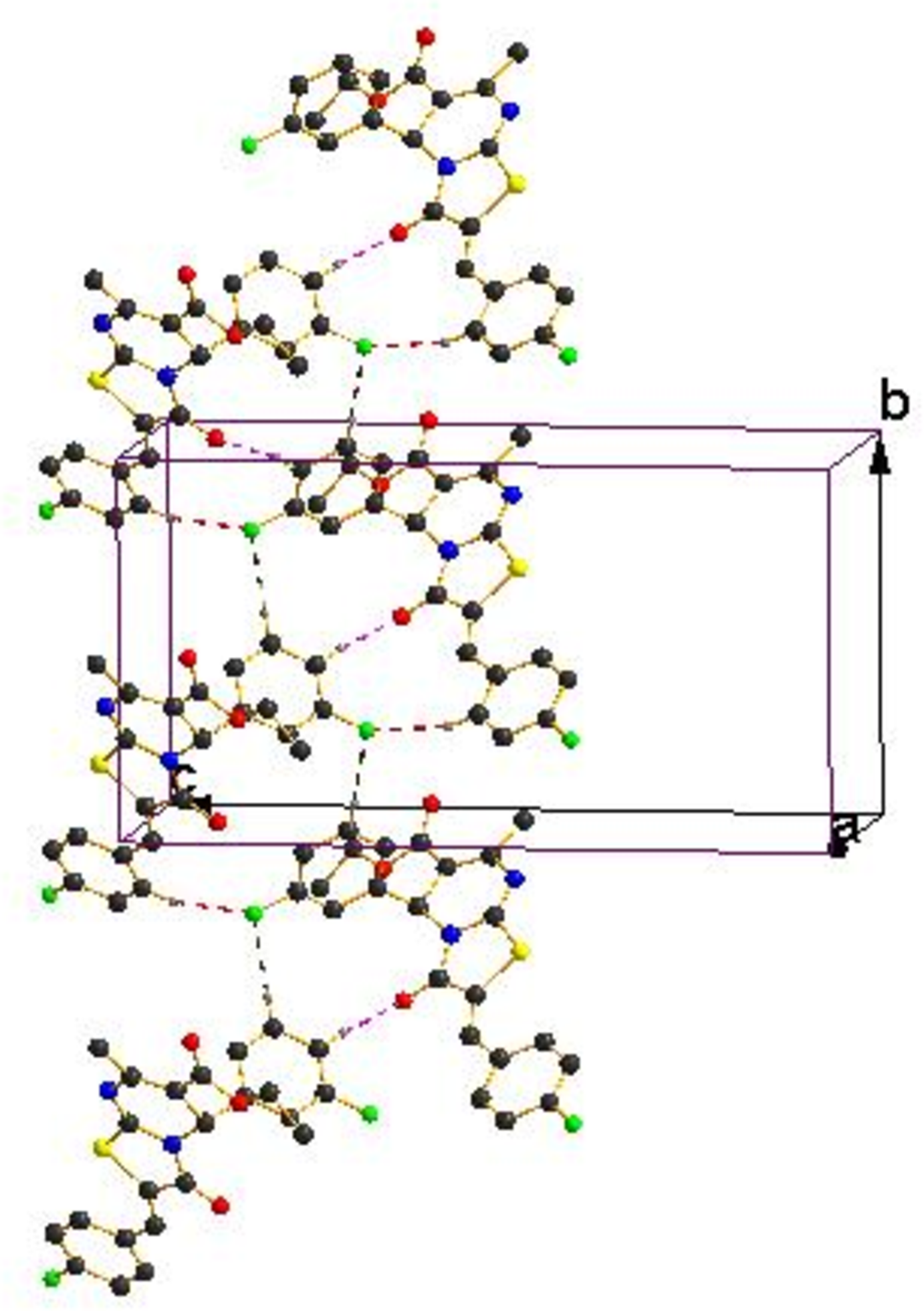

Supplement: Supplementary file 5 [file e-70-o1187-fig2.tif]
